# Meningococcus B

**DOI:** 10.1186/1824-7288-41-S2-A9

**Published:** 2015-09-30

**Authors:** Gianni Bona, Matteo Castagno

**Affiliations:** 1 Clinica Pediatrica, Department of Scienze della Salute, University of Piemonte Orientale, Italy

## 

Between 13 different serogroups of *N. meningitidis*, identified according to the capsular polysaccharide's antigenic structure, only 5 (A, B, C, W-135 and Y) are clinically relevant and responsible for 90% cases of meningococcal invasive disease. In Italy, serogroup B is the principal cause of invasive meningococcal disease [1].

There are two types of tetravalent vaccines for serogroups A, C, W-135 and Y, represented by polysaccharide and conjugate vaccines. However, only recently it has been possible to develop a vaccine against meningitis B with the “Reverse vaccinology” technique: after identifying more than 600 proteins as immunological potential targets, 91 have been selected (expressed on the outer capsule). Only 28 of them could induce a bactericidal activity and, among these, 3 have been selected: fHbp, NHBA and NadA, which were able to stimulate an antibody protection, in addition to proteins OMV (4CMenB)[2].

In November 2012, the multicomponent vaccine 4CMenB was approved from the European Medicines Agency (EMA). In January 2013, the European Commission authorized the marketing of the new vaccine 4CMenB (Bexsero®), addressed to immunization from two months of age [3,4].

In Italy this authorization is implemented by AIFA determines of May 27, 2013.

Moreover, in 2014 in the US, a new anti-meningococcal bivalent vaccine was approved: Trumenba® (Pfizer). Currently, 4CMenB has been authorized in 12 countries worldwide, the largest of which are Australia and Canada. Already 151,800 subjects have received at least one dose of vaccine in 18 countries worldwide.

In Italy, Basilicata has been the first region that implemented 4CMenB in its vaccination schedule, with resolution in 24 February 2014. A few months later also Puglia, Liguria, Tuscany, Veneto, Sicily, Friuli, Bolzano and Calabria have introduced the meningococcal B vaccine, and other regions are ready to follow them.

In the new Italian vaccination calendar, proposed by SLTL, FIMP and SIP, 4CMenB has been prepared in a 4-doses schedule, in consideration of the higher incidence of the infection among the first 4-6 months of life (first cycle based on three doses in the first year, starting from the 75th day of life, and a forth dose at 13-15 months of age) [1].
